# In-Plant Validation of Novel On-Site Ozone Generation Technology (Bio-Safe) Compared to Lactic Acid Beef Carcasses and Trim Using Natural Microbiota and Salmonella and *E. coli* O157:H7 Surrogate Enumeration

**DOI:** 10.3390/foods10051002

**Published:** 2021-05-04

**Authors:** Diego E. Casas, David A. Vargas, Emile Randazzo, Dan Lynn, Alejandro Echeverry, Mindy M. Brashears, Marcos X. Sanchez-Plata, Markus F. Miller

**Affiliations:** 1 International Center for Food Industry Excellence, Department of Animal and Food Sciences, Texas Tech University, Lubbock, TX 79409, USA; diego.casas@ttu.edu (D.E.C.); Andres.vargas@ttu.edu (D.A.V.); Alejandro.echeverry@ttu.edu (A.E.); mindy.brashears@ttu.edu (M.M.B.); mfmrraider@aol.com (M.F.M.); 2 Nebraska Beef Ltd., Omaha, NE 68107, USA; edazzo@nbeef.com; 3 BioSecurity Technology, Omaha, NE 68107, USA; dan@biosecuritytechnology.com

**Keywords:** *Salmonella* spp., *E. coli*, pathogen surrogates, ozone intervention, beef, beef trim

## Abstract

The purpose of this study was to evaluate the antimicrobial efficacy of an aqueous ozone (Bio-Safe) treatment and lactic acid solutions on natural microbiota and *E. coli* O157:H7 and Salmonella surrogates on beef carcasses and trim in a commercial beef processing plant. For every repetition, 40 carcass and 40 trim swabs (500 cm^2^) were collected. Samples were taken using EZ-Reach^TM^ swabs, and plated into aerobic plate count (APC), coliform, and *E. coli* Petrifilm^TM^ for enumeration. In addition, a five-strain cocktail (MP-26) of *E. coli* surrogates was inoculated onto trim. For every trim surrogate repetition, 30 trim pieces were sampled after attachment and after ozone intervention. Samples were diluted and counts were determined using the TEMPO^®^ system for *E. coli* enumeration. Ozone and lactic acid interventions significantly reduced (*p* < 0.003) bacterial counts in carcasses and trim samples. Moreover, lactic acid further reduced APC and coliforms in trim samples compared to ozone intervention (*p* < 0.009). In the surrogate trials, ozone significantly reduced (*p* < 0.001) surrogate concentration. Historical data from the plant revealed a reduction (*p* < 0.001) of presumptive *E. coli* O157:H7 in trim after a full year of ozone intervention implementation. The novel technology for ozone generation and application as an antimicrobial can become an alternative option that may also act synergistically with existing interventions, minimizing the risk of pathogens such as Salmonella and *E. coli* O157:H7.

## 1. Introduction

Ever since the U.S. Department of Agriculture Food Safety and Inspection Service (FSIS) declared *E. coli* O157:H7 and Shiga toxin-producing *E. coli* (STEC) as adulterants in non-intact beef [1], the North American beef industry has continuously evaluated and implemented the use of antimicrobial interventions during beef harvest and processing. In addition to STECs, Salmonella presence on beef has also been identified as a significant threat to public health and an economic burden to the beef industry. Just recently, Salmonella has been linked to foodborne outbreaks and millions of pounds of ground beef have been recalled for risk of Salmonella presence in ground beef [2,3]. Despite the industry efforts to implement proper sanitary dressing procedures, best practices, and use of antimicrobial interventions, hides, and endogenous extra-intestinal sources of pathogens can contaminate beef carcasses [4]. Not one single intervention has been found to render a beef product completely safe. Thus, a multi-hurdle approach of a series of targeted antimicrobial interventions can more effectively reduce the risk of possible contamination through the slaughter process, consequently improving the microbial quality of carcasses [5]. A combination of physical and chemical interventions on beef carcasses and products may prove to be more effective than applying the same intervention at multiple stages of the slaughter and processing lines [6]. Therefore, exploring suitable and effective antimicrobial intervention alternatives may prove to be beneficial when finding synergies with already existing and implemented interventions that will further contribute to improving beef safety.

BioSecurity Technology has developed a novel ozone intervention known as Bio-Safe™ cleaning solution [7]. Aqueous ozone’s oxidation-reduction potential grants it the capacity to be used as a disinfectant by causing cell lysis and damaging nucleic acids [8]. Although the antimicrobial properties of ozone are well documented [9], previous studies assessing ozone’s potential as an intervention in beef carcasses have had contradictory results, where some have significantly reduced *E. coli* O157:H7 concentration whereas others have found no significant difference than water wash (28 °C) treatments [10,11]. Whether an intervention works in a laboratory environment or not, does not determine its feasibility or effectiveness in the beef processing plant environment, and therefore in-plant validation studies must be conducted in a particular commercial beef processing plant to assess its real effectiveness. Lactic acid is listed in FSIS Directive 7120.1 as a safe and suitable ingredient in the production of meat products. It may be used on beef subprimals at the amount of 2 to 5 percent solution not to exceed 55 °C (131 °F). The same Directive states that ozone is safe for use on all meat products per current industry standards. There are no labeling requirements on these single-ingredient items providing the use of the substance is consistent with the FDA’s definition of a processing aid, and the application on meat meets all water retention requirements of 9 CFR 441.10.

Because foodborne pathogens should not be introduced into the beef processing environment under any circumstance, *E. coli* O157:H7 and Salmonella surrogates have been developed to validate antimicrobial interventions in commercial beef processing plants without compromising safety [12]. In this study, we hypothesize that the aqueous ozone intervention will significantly reduce indicator microorganisms naturally present in beef carcasses and trim in a commercial beef processing plant environment. Furthermore, we also evaluated if this intervention significantly reduces an *E. coli* O157:H7 and Salmonella surrogate-cocktail inoculated in beef trim in a commercial beef processing facility.

## 2. Materials and Methods

### 2.1. Intervention Parameters

Lactic acid operation parameters as applied in the plant used for this study included a spray treatment solution with a temperature of 110–130 °F (43–55 °C), at 2–5% lactic acid concentration with a spray pressure ≥15 psi. Bio-safe by BioSecurity Technology (Ozone) intervention operating parameters included ozone generators which utilize oxygen molecules from the air (O_2_) and pass them through a corona field, splitting them into single atoms of oxygen (O_1_). These atoms combine with an O_2_ molecule to form a molecule of O_3_ (Ozone). After the intervention and immediate reaction with organic matter, it turns back into oxygen, leaving no harmful byproducts or residuals according to manufacturer’s description and proprietary technology developed. Oxidation-Reduction Potential (ORP) instrumentation is used to monitor and control the reactivity and effectiveness of the sanitizing power of ozonated water. The aqueous ozone treatment spray had incoming water maintained at 50–75 °F (10–24 °C), the concentration was 1.5–2.3 ppm and the ORP was measured by an in-line meter between 700 and 900 mV with a spray pressure of ≥20 psi. Ozone application consisted of a multiple hurdle carcass intervention system with three treatment cabinets using the following specifications: 52 spray nozzles delivering 24.6 gpm with 5 s treatment time, 62 spray nozzles delivering 34.6 gpm with 5 s contact time, and 36 spray nozzles delivering 13.6 gpm with 20 s contact time for each cabinet, respectively. The cumulative application used was 72.8 gpm with a total of 30 s contact time in carcasses. Moreover, the trim ozone intervention consisted on one treatment cabinet with 44 nozzles delivering 12.8 gpm with 18 s contact time.

### 2.2. Evaluation of Natural Microbiota on Carcass and Trim

For each repetition, in one production day, samples were randomly collected before and after treatment. A total of 20 carcasses were sampled before and after the final intervention. Of these carcasses, 10 were treated with lactic acid intervention and 10 with the ozone treatment intervention. Samples were taken before intervention at the harvest floor and after intervention at the hot box, for a total of 40 carcass swabs per repetition. The next day, trim was fabricated from the carcasses that were treated with the ozone intervention and lactic acid intervention, traced, and separated into different trim combos. Ten representative pieces of trim that came from the carcasses with the ozone intervention and 10 pieces of trim that came from carcasses with the lactic acid intervention were sampled before and after the trim intervention. The selected carcasses and trim were sampled on an area of 500 cm^2^ using 25 mL buffered peptone water (BPW) EZ-Reach^TM^ swabs (World Bioproducts, Mundelein, IL, USA). Carcasses were sampled on the foreshank area, trim was sampled on several points until reaching approximately the target area of 500 cm^2^. Samples were collected by Texas Tech University (TTU) trained personnel. Swab samples were immediately chilled and shipped overnight to the ICFIE-TTU Food Microbiology laboratory for microbiological analysis. Swab samples were homogenized in a stomacher (Model 400 circulator, Seward, West Sussex, UK) at 230 rpm for 1 min. Next, samples were serially diluted in 9 mL BPW (Millipore Sigma, Danvers, MA, USA) tubes and plated to determine total aerobic plate counts (APC), coliform counts, and *E. coli* counts using 3M^TM^ Petrifilm^TM^ (Saint Paul, MN, USA) plates. The counts of each sample were determined and converted to Log CFU/cm^2^ for carcasses and Log CFU/sample for trim samples before statistical analysis. A total of six repetitions were conducted.

### 2.3. Salmonella and E. coli O157:H7 Surrogate Inoculation in Trim

#### 2.3.1. Nonpathogenic Cocktail Preparation

Five non-pathogenic American Type Culture Collection (ATCC) Salmonella and *E. coli* O157:H7 surrogate strains were selected for this section of the study. These strains of non-virulent *E. coli* (BAA 1427, 1428, 1429, 1430, and 1431), when used as a cocktail, have been previously shown to mimic Salmonella and *E. coli* O157:H7 antimicrobial intervention behavior [13,14,15,16]. The use of surrogate strains to validate interventions in plant environments has been previously discussed and at times encouraged by FSIS USDA, which has allowed the use of such non-pathogenic surrogates with appropriate precautions [12]. The surrogate strains were independently propagated in a food grade biological safety level I (BSL-I) laboratory at TTU. Each ATCC strain was retrieved from a −80 °C freezer, separately transferred into 4 mL brain heart infusion (BHI; Becton, Dickinson and Company, Franklin Lakes, NJ, USA) tubes, and incubated at 37 °C for 18–24 h. Next, overnight enriched tubes were screened for *E. coli* O157:H7 and Salmonella presence using BAX^®^ real-time *E. coli* O157:H7 Exact and Salmonella assays (Hygiena, Wilmington, DE). After found negative for both pathogen screenings, 500 µL of each enriched surrogate broth was transferred into 49.5 mL BPW tube and cleared to be used for the challenge study. Then, all five tubes were decanted onto a sprayer and mixed. The bottle sprayer was then used for trim target inoculation of 5–6 LogCFU/cm^2^.

#### 2.3.2. Trim Inoculation and Quantification

For each repetition, chuck and shank trim were randomly selected for inoculation. A total of 15 pieces of chuck and 15 pieces of trim were inoculated using the sprayer. Each piece of trim was sprayed with the *E. coli* O157:H7 and Salmonella surrogate cocktail and allowed for 30 min of cell attachment while at ambient temperature. After attachment time, an area of 100 cm^2^ was sampled using a 25 mL BPW EZ-Reach^TM^ swab. Trim was next treated with the ozone treatment and immediately after intervention but before entering the production line, trim was sampled. All swabbed areas were marked with 100 cm^2^ stamped area to ensure that the same area was not sampled repeatedly. Samples were collected by TTU trained personnel and shipped overnight to the TTU Food Microbiology laboratory for microbial enumeration. Swabs were homogenized in a stomacher at 230 RPM for 1 min. *E. coli* counts were determined using the TEMPO^®^ system (Marcy-l’Étoile, France) following the manufacturer’s instructions. TEMPO^®^ cards were incubated at 35 °C for 22–28 h. *E. coli* counts were directly obtained from the TEMPO^®^ Reader and converted to LogCFU/cm^2^ before statistical analysis. A total of six repetitions were conducted.

### 2.4. Statistical Analysis

All data were analyzed using R (Version 4.0.3) Statistical analysis software to evaluate differences between lactic acid and the ozone intervention and testing for a significant reduction of microbial loads after each intervention in the natural microbiota setting was performed. A two-way ANOVA was done using intervention type (ozone and lactic acid), sampling point (before and after intervention), and their interaction as fixed effects. For the surrogate study, a two-way ANOVA was performed using trim type (chuck and shank), sampling point (before and after intervention), and their interaction as fixed effects. Post hoc analysis was done using a pairwise T-test with Bonferroni p-adjustment method for multiple comparisons. If parametric assumptions were not met, the Kruskal–Wallis test was used as a nonparametric alternative for the ANOVA, with post-hoc analysis using Wilcoxon rank-sum tests with a BH p-adjustment method for multiple comparisons. Significant differences were evaluated at the 0.05 alpha level. Historical data of *E. coli* O157:H7 presumptive positives from the commercial beef processing plant where the challenge study was conducted was shared with TTU researchers for information purposes. Chi-square comparison to identify the difference in prevalence before and after the ozone intervention application by year and on a per month basis was conducted.

## 3. Results

### 3.1. Natural Microbiota on Carcass

**Figure 1:**
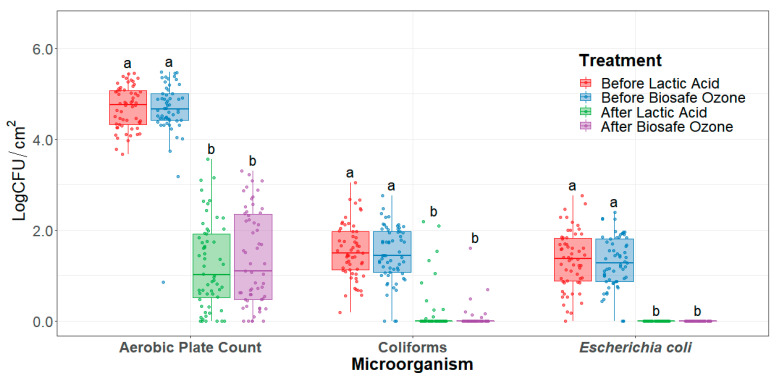
Carcass Aerobic plate count, coliform, and *Escherichia coli* counts (limit of detection < 0.05 CFU/cm^2^) before and after the application of the interventions (LogCFU/cm^2^). Horizontal line within the boxplot represents the median. The box upper and lower limit represents the interquartile range, and the bars represent 1.5xInterquartile Range. ^a,b^ Box plots with different letters within the same microorganism type represent statistical differences (*p* < 0.05).

Both lactic acid and the ozone interventions significantly reduced (*p* < 0.0001) aerobic plate counts, coliform, and *E. coli* when applied to beef carcasses (Figure 1). Aerobic plate counts on carcasses were significantly reduced on average by 3.26 Log CFU/cm^2^ and 3.83 LogCFU/cm^2^ after ozone and lactic acid interventions, respectively. Coliform counts on carcasses were significantly reduced on average by 1.42 Log CFU/cm^2^ and 1.37 Log CFU/cm^2^ after ozone and lactic acid interventions, respectively. Likewise, *E. coli* counts on beef carcasses were significantly reduced by 1.29 LogCFU/cm^2^ and 1.35 LogCFU/cm^2^ after ozone and lactic acid intervention, respectively. Significant reduction of *E. coli* to undetectable levels was achieved after lactic acid and ozone interventions on beef carcasses. For each microorganism, there were no statistical differences in microbial populations between any of the two interventions.

### 3.2. Natural Microbiota on Trim

**Figure 2:**
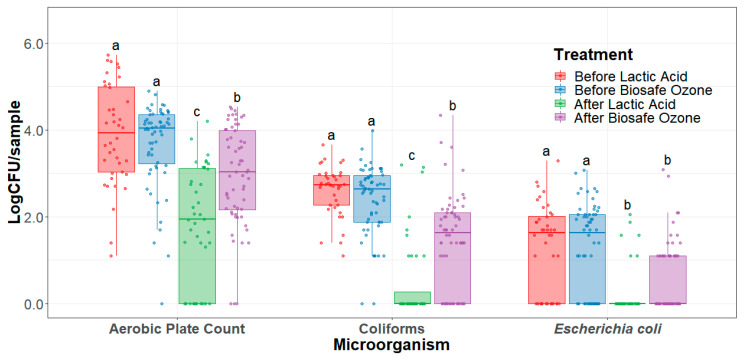
Trim aerobic plate count, coliforms, and *Escherichia coli* counts (limit of detection < 0.05 CFU/cm^2^) before and after the application of the interventions (Log CFU/sample). Horizontal line within the boxplot represents the median. The box upper and lower limit represents the interquartile range, and the bars represent 1.5xInterquartile Range. ^a,b^ Box plots with different letters within the same microorganism type represent statistical differences (*p* < 0.05).

Coliforms and *E. coli* counts on the trim were substantially low when analyzed on a per cm^2^ basis. When transformed to Log CFU/cm^2^ for statistical analysis, most counts were below 1 CFU/cm^2^, therefore resulting in negative Log CFU/cm^2^ counts, making analysis and visualization more difficult. Thus, an analysis on a per sample (Log CFU/500 cm^2^) basis was made to assess the effectiveness of the interventions. This conversion was achieved by multiplying the Log CFU/cm^2^ by 500 cm^2^ of area sampled, resulting in Log CFU/500 cm^2^ which is equivalent to Log CFU/sample. On trim, both lactic acid and the ozone interventions significantly reduced (*p* < 0.003) aerobic plate counts, coliform, and *E. coli* when applied to trim (Figure 2). Moreover, lactic acid greatly reduced (*p* < 0.009) aerobic plate count and coliforms when compared to ozone. Aerobic plate counts on trim were significantly reduced on average by 0.74 Log CFU/sample and 2.08 Log CFU/sample after ozone and lactic acid interventions, respectively. Coliform counts on trim were significantly reduced on average by 0.93 Log CFU/sample and 2.13 Log CFU/sample after ozone and lactic acid interventions, respectively. Moreover, *E. coli* counts on beef trim were significantly reduced on average by 0.67 Log CFU/ sample and 1.08 Log CFU/sample after ozone and lactic acid interventions, respectively.

**Figure 3:**
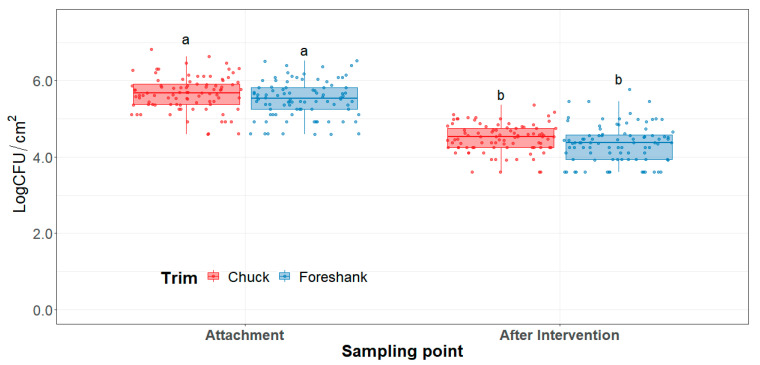
*Escherichia coli* surrogate attachment levels and after intervention counts (limit of detection < 4 CFU/cm^2^) on LogCFU/cm^2^ basis. Horizontal line within the boxplot represents the median. The box upper and lower limit represents the interquartile range, and the bars represent 1.5xInterquartile Range. ^a,b^ Box plots with different letters represent statistical differences (*p* < 0.05).

Since trim natural microbiota encountered in coliforms and *E. coli* was substantially low, authors decided to inoculate *E. coli* O157:H7 and Salmonella surrogates on the trim and apply the ozone intervention to assess its efficacy. For both trim types, the ozone intervention significantly reduced (*p* < 0.0001) *E. coli* O157:H7 and Salmonella surrogate cocktail counts (Figure 3). Initial inoculation attachment was on average 5.67 Log CFU/cm^2^ and 5.52 Log CFU/cm^2^ for chuck and foreshank trim, respectively. *E. coli* cocktail attachment was well within target inoculation of 5–6 Log CFU/cm^2^. On average, counts were reduced by 1.17 Log CFU/cm^2^ after the ozone intervention. Reduction between trim types was similar (*p* = 0.18). Consequently, the intervention efficacy is expected to be the same when applied to different trim types.

**Figure 4:**
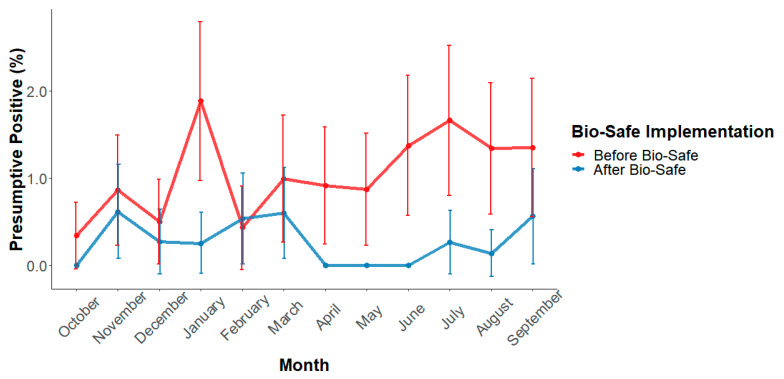
In-plant monthly Presumptive positive rate of *E. coli* O157:H7 in beef trim before and after implementation of the ozone intervention (N = 19,048). ^I^ Error bars represent 95% confidence intervals of the monthly incidence.

In the beef processing plant, the use of the ozone intervention was implemented on 11 October 2019. Chi-square analysis comparing the year prior (1.06%, 102/9,609) to implementation of Biosafe ozone intervention and the year after (0.26%, 25/9,439) implementation indicates statistical difference (*p* < 0.0001) in the percentage of presumptive positive rates of *E. coli* O157:H7 in trim per year. A month-by-month comparison can be observed in Figure 4. The year before implementation of the ozone intervention presented a 4.1 times greater incidence of presumptive *E. coli* O157:H7 than the year after implementation, indicating a potential 75.5% reduction of presumptive *E. coli* O157:H7 presence in trim.

## 4. Discussion

The ozone intervention in carcasses significantly reduced indicator microorganisms studied in the commercial beef processing plant environment. This reduction was equivalent in magnitude to the reduction observed by using a final lactic acid carcass wash. The processing plant that allowed this study to be conducted, used 82 °C (180 °F) hot carcass wash prior to the lactic acid wash as their usual final harvest intervention before the carcasses entered the hot box. For this study, they left the hot water wash on and switched the lactic acid spray with the aqueous ozone treatment to evaluate the effect of ozone compared to that achieved with the use of lactic acid. Consequently, it can be observed that the multiple hurdle approach of using ozone after a hot water wash has equivalent reduction of APC, coliforms, and *E. coli* compared to using lactic acid after a hot water wash. Minimal sampling requirements to demonstrate process control in beef slaughter operations published by the FSIS require one generic *E. coli* sample for every 300 head of cattle harvested. A negative result is the acceptable outcome, but if in 13 subsequent generic *E. coli* tests there are more than three samples between 1 and 100 CFU/cm^2^, the commercial processing plant fails the performance standards [17]. In this study, *E. coli* cell count was below the detection limit (<0.05 CFU/cm^2^) after both final carcass interventions. Thus, the facility passed the performance standards and can demonstrate appropriate process control while using lactic acid or ozone interventions.

Ozone in an aqueous solution has been used in the past as a possible antimicrobial intervention in beef. Some studies have reported no significant reduction compared to a 28 °C water wash, whereas others have observed a significant reduction of 1.46 LogCFU/cm^2^ of *E. coli* O157:H7 compared to 0.60 LogCFU/cm^2^ reduction of water spray chill and a reduction of APC of 0.99 LogCFU/cm^2^ [10,11]. In this study, a reduction of APC of 3.26 LogCFU/cm^2^ was observed after hot water wash and ozone treatment. A multiple hurdle approach in the commercial plant environment is followed to more effectively eliminate pathogen presence in beef products [18,19]. Therefore, different interventions can act synergistically and more effectively to reduce the microbial load of beef in a commercial processing plant. Moreover, the recent development of an enhanced ozone technology and techniques to increase ozone half-life and reactivity in aqueous solution may increase the efficacy of ozone interventions in beef as observed in this study.

When comparing the ozone intervention against the lactic acid intervention in beef trim, we assessed the individual effect that the intervention has on trim. It is worth noting that the analysis in trim was done on a per-sample basis instead of a per-cm^2^ basis due to substantially low coliform and *E. coli* presence in commercial samples. In this trim study, lactic acid further reduced APC and coliform counts compared to the aqueous ozone treatment. However, similar reductions were observed in generic *E. coli* when comparing both treatments. Lactic acid has been known to have a residual effect in the reduction of microbial load, where significant reductions in indicator microorganisms can be seen even after 12 days of treatment [20]. Contrastingly, ozone interventions have not yet been observed to have a residual effect in beef, since it is unstable and breaks down into oxygen shortly after generation and reaction with organic materials. Further research must be conducted to assess differences in shelf-life effects that ozone interventions may have in beef over extended storage times.

Generic *E. coli* has historically been used by processing plants to verify process control. The hazard analysis and critical control points system final rule of 1996 required generic *E. coli* testing [21]. *E. coli* presence is important to assess in beef because it is an indicator of fecal contamination as it is commonly found in the cattle gastrointestinal tract and hides. The gastrointestinal tract of cattle is also a possible reservoir of foodborne pathogens such as Salmonella and *E. coli* O157:H7 [17]. Therefore, if *E. coli* is found in beef, the risk of having Salmonella or pathogenic *E. coli* presence is likely to increase. In the trim sampled, over 90% of the trim had < 1 CFU/cm^2^ of *E. coli*. Thus, to further validate the efficacy of the ozone treatment, the authors decided to conduct a Salmonella and *E. coli* O157:H7 surrogate inoculation study on the trim inside a commercial beef processing plant, to take into account the effects of commercial processing operations and actual equipment.

In the surrogate inoculation trial, ozone intervention significantly reduced the concentration of the *E. coli* cocktail. Foreshank and chuck trim were chosen as the “worst case scenario” for this section as, historically, these are the two types of trim that the commercial beef processing plant had more frequently found presumptive *E. coli* O157:H7 presence. These surrogates have been previously seen to mimic *E. coli* O157:H7 and Salmonella resistance to antimicrobial treatments when used as a cocktail in validation trials [13,14,15,16,22]. In some cases, reporting a slight increase in the magnitude of survival of the surrogate compared to Salmonella or *E. coli* O157:H7 for a relatively higher margin of safety. Thus, it can be inferred that the survival of the pathogens would be less than the one encountered with the surrogates. The surrogates are more on the conservative end of possible reduction since some of these strains might be slightly more resistant to an antimicrobial intervention than the actual pathogens [13,16]. In this context, the ozone intervention can significantly reduce *E. coli* O157:H7 and Salmonella average concentration by at least 1.17 LogCFU/cm^2^, with further reductions potentially possible if subsequent sequential applications are considered and surface contact is enhanced. Furthermore, the antimicrobial intervention may cause sublethal injuries in cells that may hinder their ability to grow in selective media. Even though the samples were kept at refrigerating temperatures for approximately 24 h prior to processing in BPW while being shipped to the laboratory, bacteria may have not completely recovered from the intervention. However current sampling and quantification protocols used by the North American beef industry for *E. coli* follow quantification in selective media.

Historical data shared by the plant indicates a significant improvement since the implementation of the ozone intervention in the commercial facility. The year before ozone implementation, 102 lots of trim resulted in presumptive positive for *E. coli* O157:H7. After a year of ozone implementation, the plant observed a 75.5% reduction in positives, having only 25 presumptive positive lots. The improvement translates into a significant economic gain as substantially fewer lots of trim had to be disposed of or rerouted to fully cooked products at lower values. Ozone is known to have antimicrobial properties through direct oxidation of the cell wall resulting in cell lysis; however, it can also considerably damage DNA and produce reactions with oxygen radical by-products during its breaking down process [8]. Current methods for *E. coli* O157:H7 detection in beef, have screening procedures that use quantitative PCR for detection of a particular gene encoded in the DNA of the pathogen of interest [23]. In the multiple hurdle intervention setting, bacteria have been affected by a series of antimicrobial interventions, such as hot carcass washes, organic acid washes, carcass trimming, steam vacuuming, among others. By the time carcasses reach the chilling rooms, they have potentially undergone at least 2–4 antimicrobial interventions possibly reducing bacterial loads below detection limits, as it can be observed in coliform and *E. coli* counts in carcasses after interventions evaluated in this study. At that point, an ozone intervention may be able to further reduce bacterial concentration through cell lysis or other mechanisms; such as DNA damaging that has been reported [24,25] and ozone could have accessibility due to the synergistic effect on the bacterial membrane, that may be weakened from the prior antimicrobials used in the facility When cells undergo such damage, their proliferation becomes hindered under stressful conditions, such as refrigeration storage and distribution, enhancing beef safety in the value chain. Ozone’s capacity for DNA degradation may be causing mutations in the bacterial genome rendering bacteria harmless and target genes of the real-time PCR screening procedures undetectable [24]. More research is needed to confirm cell damage and viability after the application of sequential ozone treatments, but these findings provide evidence that the aqueous ozone intervention evaluated in this study may play a significant role in controlling pathogen contamination in beef carcasses and trim.

## 5. Conclusions

The novel proprietary technology used to produce the high concentration, and stable reactivity of the aqueous ozone solution proved promising for the reduction of *E. coli* O157:H7 detection and indicator levels in beef. The findings encountered in this study indicate that the ozone intervention is not only effective but similar in performance to lactic acid in reducing bacterial load on carcasses and trim which will improve beef safety, therefore validating its use in the beef processing environment as an effective antimicrobial intervention. Bacterial surrogate studies become of utmost importance when trying to validate interventions in a commercial processing plant setting. They more accurately represent the specific effects that the antimicrobial intervention will have against pathogens they represent in a given environment, without compromising food safety. The evaluation of in-plant data for comparative purpose of intervention schemes gives additional support to the effectiveness of this technology, with ongoing control exerted over different seasons and processing months. Further research into multiple hurdle intervention interactions must be conducted to design the most effective ways of mitigating pathogen presence and ensure beef safety.

## Data Availability

Data available on request from the corresponding author. The data are not publicly available due to privacy from the beef processing partner that allowed the project to be conducted within their beef processing environment.
